# Effects of Robotic Postural Stand Training with Epidural Stimulation on Sitting Postural Control in Individuals with Spinal Cord Injury: A Pilot Study

**DOI:** 10.3390/jcm13154309

**Published:** 2024-07-24

**Authors:** Enrico Rejc, Simone Zaccaron, Collin Bowersock, Tanvi Pisolkar, Beatrice Ugiliweneza, Gail F. Forrest, Sunil Agrawal, Susan J. Harkema, Claudia A. Angeli

**Affiliations:** 1 Tim and Caroline Reynolds Center for Spinal Stimulation, Kessler Foundation, 1199 Pleasant Valley Way, West Orange, NJ 07052, USA; gforrest@kesslerfoundation.org (G.F.F.); cangeli@kesslerfoundation.org (C.A.A.); 2 Department of Medicine, University of Udine, P.le Kolbe 4, 33100 Udine (UD), Italy; simone.zaccaron@uniud.it; 3 Kentucky Spinal Cord Injury Research Center, University of Louisville, 220 Abraham Flexner Way, Louisville, KY 40202, USA; cdbowersock@gmail.com (C.B.); tanvi.pisolkar@gmail.com (T.P.); beatrice.ugiliweneza@louisville.edu (B.U.); susan.harkema@louisville.edu (S.J.H.); 4 Department of Neurosciences, Biomedicine and Movement Sciences, University of Verona, 37129 Verona, Italy; 5 Biomechatronics Lab, Department of Mechanical Engineering, Northern Arizona University, S San Francisco St, Flagstaff, AZ 86011, USA; 6 Department of Neurological Surgery, University of Louisville, Louisville, KY 40202, USA; 7 Department of Physical Medicine and Rehabilitation, Rutgers New Jersey Medical School, Newark, NJ 07103, USA; 8 Department of Mechanical Engineering, Columbia University, 220 S. W. Mudd Building, 500 West 120th Street, New York, NY 10027, USA; sunil.agrawal@columbia.edu; 9 Department of Rehabilitation and Regenerative Medicine, Columbia University, New York, NY 10032, USA

**Keywords:** epidural stimulation, spinal cord injury, postural control, sitting, stand training

## Abstract

(1) **Background**. High-level spinal cord injury (SCI) disrupts trunk control, leading to an impaired performance of upright postural tasks in sitting and standing. We previously showed that a novel robotic postural stand training with spinal cord epidural stimulation targeted at facilitating standing (Stand-scES) largely improved standing trunk control in individuals with high-level motor complete SCI. Here, we aimed at assessing the effects of robotic postural stand training with Stand-scES on sitting postural control in the same population. (2) **Methods**. Individuals with cervical (*n* = 5) or high-thoracic (*n* = 1) motor complete SCI underwent approximately 80 sessions (1 h/day; 5 days/week) of robotic postural stand training with Stand-scES, which was performed with free hands (i.e., without using handlebars) and included periods of standing with steady trunk control, self-initiated trunk and arm movements, and trunk perturbations. Sitting postural control was assessed on a standard therapy mat, with and without scES targeted at facilitating sitting (Sit-scES), before and after robotic postural stand training. Independent sit time and trunk center of mass (CM) displacement were assessed during a 5 min time window to evaluate steady sitting control. Self-initiated antero-posterior and medial-lateral trunk movements were also attempted from a sitting position, with the goal of covering the largest distance in the respective cardinal directions. Finally, the four Neuromuscular Recovery Scale items focused on sitting trunk control (Sit, Sit-up, Trunk extension in sitting, Reverse sit-up) were assessed. (3) **Results**. In summary, neither statistically significant differences nor large Effect Size were promoted by robotic postural stand training for the sitting outcomes considered for analysis. (4) **Conclusions**. The findings of the present study, together with previous observations, may suggest that robotic postural stand training with Stand-scES promoted trunk motor learning that was posture- and/or task-specific and, by itself, was not sufficient to significantly impact sitting postural control.

## 1. Introduction

Motor complete, high-level spinal cord injury (SCI) disrupts the communication between supraspinal and spinal centers, leading to the paralysis of lower limbs and the severe impairment of trunk control. Upright sitting postural control requires the proper activation of trunk muscles, which is severely impacted by injuries above cervical (C) 4 level; it is also significantly affected by lesions at the lower cervical and upper thoracic levels [1,2,3]. This condition results in compromised trunk stability, which has direct implications for higher risks of falling (and related sequelae), even from a quiet seated position [4,5,6], and for impaired performance of activities of daily living, such as the control of upper limb movements and transfer ability, among others [7,8]. Trunk mobility, including the ability to reach toward different directions from a seated position, is also critically affected by severe, high-level SCI [9,10,11]. Improvement of trunk postural control, and particularly trunk stability, is recognized as one of the key rehabilitative goals as well as one of the top priorities indicated by individuals living with a SCI [12].

In the past decade, the implementation of spinal cord epidural stimulation (scES) with individual- and task-specific parameters, combined with activity-based training, has shown remarkable potential for the recovery of standing, walking, and voluntary leg movements in individuals with chronic, clinically motor complete SCI [13,14,15,16,17]. A smaller amount of evidence also supports the view that spinal cord stimulation applied to SCI individuals that are seated can promote improved trunk stability and posture [4,18] as well as a better dynamic sitting postural control during reaching tasks [10,11]. Spinal cord transcutaneous stimulation applied during activity-based training focused on trunk control from seated and supine positions was also found effective for improving static and dynamic sitting balance in individuals with complete tetraplegia [11].

A recent series of proof-of-principle studies by our group showed the feasibility and potential effectiveness of using a robotic upright stand trainer (RobUST) [19] in individuals with chronic motor complete SCI for the assessment and practice of standing postural control [20,21]. We reported that individuals with motor complete SCI receiving scES to facilitate standing (Stand-scES) generated distinct lower limb postural responses to controlled trunk perturbations. These responses, characterized by kinetic, kinematic, and EMG outcomes, were generally larger and more appropriate when the participants’ hands were free (i.e., unconstrained) rather than placed on a fixed handlebar for self-balance assistance [20]. These observations contributed to define the characteristics of a novel robotic postural stand training with Stand-scES. This training included constant robotic assistance at the pelvis and participants’ free hands, challenging standing upright posture primarily by targeting the trunk’s steady and dynamic control. Importantly, the six individuals with high-level SCI who underwent approximately 80 sessions of robotic postural training with Stand-scES showed remarkable re-emergence and/or large improvement of independent trunk control during standing. In particular, the control of steady standing posture and dynamic tasks such as self-initiated trunk movements and upper limb reaching, performed with free hands and without any assistance at the trunk, was substantially enhanced after training [21].

Therefore, in this study we assessed whether the improvements in standing trunk control promoted by robotic postural stand training with Stand-scES may also lead to enhanced trunk control during stable and dynamic sitting tasks. We hypothesized the positive effects of robotic postural stand training on sitting trunk control.

## 2. Materials and Methods

### 2.1. Participants

The participants of this study were six individuals with chronic, cervical, or high-thoracic motor complete SCI (Table 1) who (i) were already implanted with an scES unit [22] for the recovery of motor function and also met the following inclusion criteria: (ii) at least 18 years of age; (iii) having non-progressive SCI; (iv) at least 2 years post injury; (v) having a stable medical condition; (vi) unable to stand independently without scES and upper limb support. Exclusion criteria for this study were (i) being ventilator-dependent; (ii) having untreated painful musculoskeletal dysfunction, fracture, or pressure sores; (iii) having untreated psychiatric disorder or ongoing drug abuse; (iv) having cardiovascular, respiratory, bladder, or renal disease unrelated to SCI; (v) being pregnant at the time of enrollment or planning to become pregnant during the time course of the study. This open-label, single-group pilot study was performed at the Kentucky Spinal Cord Injury Research Center, University of Louisville. Prior to the beginning of this study, research participants had practiced sitting postural control with Sit-scES, including stable tall sitting and trunk movement tasks, on their wheelchair and/or on a standard therapy mat for a total duration ranging between 3 and 268 h (Table 1) as part of other experimental protocols. Comprehensive data on Sit-scES usage for B07 were not available.

The research participants signed an informed consent for scES implant and application, activity-based training, physiological monitoring studies, and publication of the related results. This study was approved by the University of Louisville Institutional Review Board (IRB #17.1024) and was conducted according to the Declaration of Helsinki.

### 2.2. Spinal Cord Epidural Stimulation Implant and Parameters

During the scES surgical implantation procedure, a midline bilateral laminotomy was performed, typically at the L1-L2 disk space. An electrode array with 16 contacts (Specify 5-6-5 lead, Medtronic, Minneapolis, MN, USA) was placed into the epidural space at the midline. Electrophysiological mapping was performed after initial placement to optimize the location of the paddle electrode based on evoked responses recorded from surface EMG electrodes (Motion Lab Systems, Baton Rouge, LA, USA) placed over representative lower limb muscles. After the final placement of the electrode array, the electrode lead was tunneled subcutaneously and connected to the neurostimulator (Medtronics, Intellis in participants A96, A101, A82, B45 and B07; RestoreADVANCED in participant B23).

In this study, tonic individual-specific scES parameters were implemented to facilitate sitting postural control (Sit-scES, Figure S1) during the related assessments. Sit-scES electrode configuration, stimulation frequency, and pulse width remained constant for the sitting assessments performed before and after robotic postural stand training. Research participants received stimulation parameters that promoted standing (Stand-scES, Figure S2) during robotic postural stand training. The process of selection of scES parameters to facilitate sitting and standing, which was reported in previous publications by our group [15,18,23], was performed prior to the beginning of the present study because of the involvement of research participants in previous experimental protocols that included the facilitation of these motor tasks. An exception was participant B07, who did not receive proper Sit-scES parameters prior to the beginning of the present study. Because of the limited access to this participant during the COVID-19 pandemic, Sit-scES parameters were not searched for this individual, who therefore did not undergo the experimental portion comprising the sitting assessment with Sit-scES.

### 2.3. Experimental Protocol

**Figure 1:**
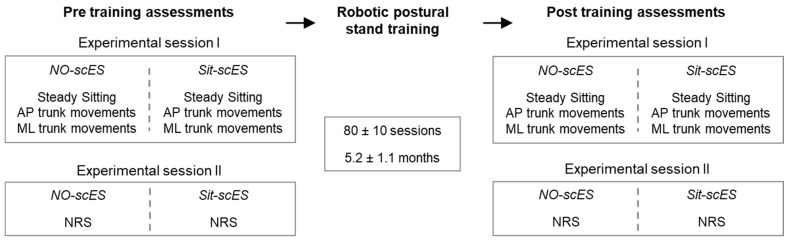
**Experimental protocol.** Two experimental sessions were performed before (Pre) and after (Post) robotic postural stand training. The portion of assessments without spinal cord epidural stimulation (NO-scES) preceded the assessments with scES to facilitate trunk control (Sit-scES).

Sitting postural control was assessed both without and with Sit-scES before the beginning (Pre training) and after the completion (Post training) of robotic postural stand training (Figure 1). Two experimental sessions were performed at each time point. One was devoted to the assessment of steady sitting and self-initiated antero-posterior (AP) and medio-lateral (ML) trunk movements. A second session was devoted to the Neuromuscular Recovery Scale assessment. In both experimental sessions, assessments were initially performed without scES and then with Sit-scES. Research participants were seated on a standard therapy mat, and research staff provided manual assistance at the trunk as needed to avoid falling if sitting postural control was not sufficient.

#### 2.3.1. Steady Sitting Assessment

During the steady sitting assessment, participants were asked to maintain a tall-sit as stable as possible, without upper limb support, for 5 min. One attempt without Sit-scES and one attempt with Sit-scES were investigated for each time point. The duration of independent sitting within the 5 min trial was determined and considered for analysis. The duration of eventual assisted sitting periods by self-assistance (i.e., participant placing their hands on the mat or on their legs) and/or the trainer’s manual assistance was also recorded and considered for analysis.

#### 2.3.2. Self-Initiated Sitting Trunk Movements

The goal of self-initiated antero-posterior (AP) and medio-lateral (ML) sitting trunk movements was to achieve the largest AP or ML trunk displacement while maintaining independent trunk control. For each trunk movement, scES condition (i.e., without or with Sit-scES) and time point, three attempts were performed, and the one with the largest trunk displacement was considered for further analysis. The need for any additional trainer’s manual assistance and/or the placement of the participant’s upper limb(s) on the mat or on their legs during the trial resulted in a failed attempt, which was not considered for further analysis. Participant A101 was the only individual requiring manual assistance to achieve steady sitting, only when Sit-scES was off. For this individual and condition only, attempts were considered failed if, during the dynamic part of the trunk movement tasks, additional manual assistance (i.e., larger level and/or toward a different direction) was needed to avoid falling because of a loss of balance control.

#### 2.3.3. Neuromuscular Recovery Scale

The neuromuscular recovery scale (NRS) was designed and validated to assess trunk and lower limb motor functions and their level of non-compensatory recovery over time [24,25]. Importantly, it is suitable and can be implemented across all AIS grades and levels of injury. The NRS is comprised of a subset of assessed executions of specific motor tasks that are performed overground, which do not entail manual facilitation by a trainer, and another subset of abilities that are assessed on a treadmill with a body weight support system. In the present study, we considered the four NRS overground items that specifically assess sitting trunk motor function, which are Sit, Sit-up, Trunk extension in sitting, and Reverse sit-up. Each NRS item is scored on a 12-point ordinal scale; the lowest score (1A) indicates that the individual is unable to perform the task, while the highest score (4C) represents the ability to perform the task as able-bodied individuals would do [26]. NRS scores were substituted with numeric values (i.e., 1A = 1 and 4C = 12) to also calculate an average score across these four trunk items.

### 2.4. Kinematic Data Acquisition and Analysis

A full-body motion capture marker set (modified Helen Hayes model—Helen Hayes Hospital, West Haverstraw, NY, USA) and an eight-camera motion capture system (Motion Analysis Corporation, Santa Rosa, CA, USA) were used to acquire kinematics. Data were acquired at 100 Hz using Cortex software Version 6.2.3.1732 (Motion Analysis Corporation, Santa Rosa, CA, USA). Identification of reflective markers was performed using the same Cortex software, and gaps were filled using linear and polynomial interpolation techniques. Ortho Trak software Version 6.6.4 (Motion Analysis Corporation, Santa Rosa, CA, USA) was then used to calculate the trunk center of mass (CM) using the 3D coordinates of shoulders and pelvis markers.

During steady sitting, trunk CM displacement was quantified in terms of root mean square (RMS) of its total distance traveled in the transverse plane. RMS is one of the CM variables that can be implemented to investigate steady postural control in able-bodied individuals as well as in those with neurological conditions [27,28,29]. During self-initiated trunk movements, trunk displacement for the AP and ML movement tasks was calculated as the AP or ML peak-to-peak distance covered by the trunk CM.

### 2.5. Robotic Postural Stand Training

**Figure 2:**
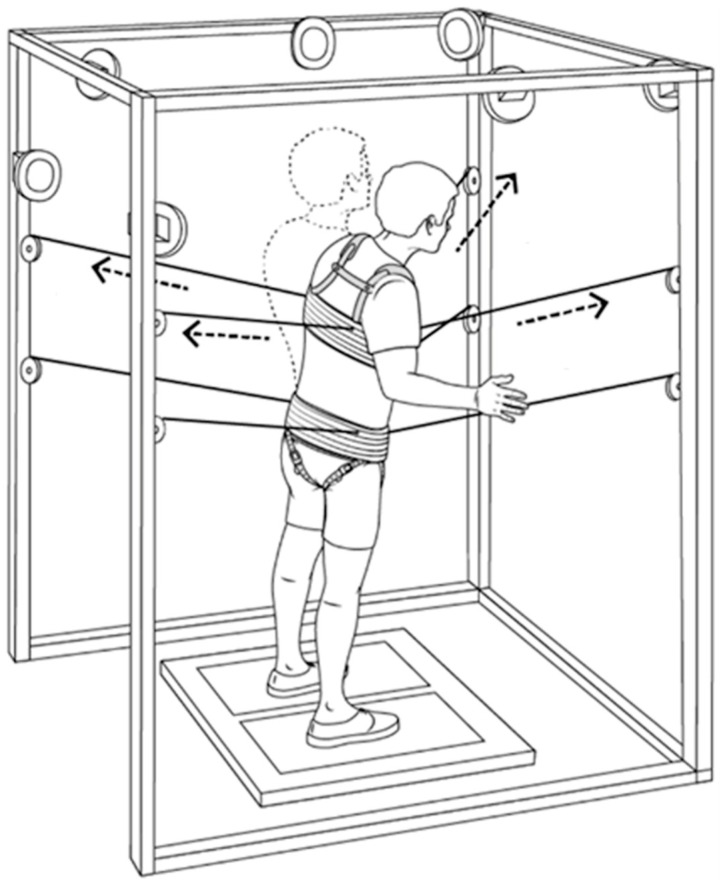
**Schematic of the Robotic Upright Stand Trainer (RobUST).** Trunk perturbation or assistance as needed forces (exemplified by the dashed arrows) are generated by mounted motors and delivered by the cables attached to the trunk harness. Pelvis motors and cables apply a constant force to assist pelvic control. Trunk kinematic data used in real time to control RobUST are collected through motion capture cameras mounted on the frame (Modified from [21]).

Research participants underwent, on average, 80 ± 10 robotic postural stand training sessions (1 h/day; 5 days/week) with Stand-scES over approximately 5.2 ± 1.1 months. The Robotic upright Stand Trainer (RobUST) implemented in the present study (Figure 2) is a cable-driven robotic device controlled by a motion capture system that can apply controlled forces at the pelvis and trunk of standing individuals by dedicated motors mounted on an aluminum frame [19]. During robotic postural stand training, RobUST applied constant force (80 N) at the pelvis to facilitate appropriate extension and pelvic tilt, with additional manual assistance from the trainer provided as necessary. During portions of each training session, RobUST applied to the trunk controlled perturbative forces toward the four cardinal directions. Conversely, during the practice of standing with steady trunk control as well as self-initiated trunk and arm movements, RobUST provided assistance as needed at the trunk of research participants. Specifically, a virtual circular boundary was programmed around the individual so that they could move freely within such a virtual boundary, while a restoring force was applied by the RobUST if the trunk moved beyond it (e.g., because of the loss of balance control), bringing the trunk back within the virtual boundary and closer to the neutral standing position [21].

Participants performed robotic postural stand training with free hands (i.e., handlebars were not used for self-balance assistance), and each training session consisted of periods of steady standing, self-initiated trunk and arm movements, and trunk perturbations delivered toward the four cardinal directions [21]. Throughout the training protocol, points of training progression included the modulation of the virtual circular boundary characteristics for trunk assistance as needed ((i) decreasing restorative force magnitude; (ii) increasing boundary radius; (iii) removing assistance as needed by disconnecting the RobUST cables from the trunk harness). The participants were also challenged throughout the training protocol by the increased complexity of the self-initiated arm movements requested ((i) lateral arm abduction; (ii) one-arm forward reaching; (iii) one-arm diagonal reaching; (iv) two-arm forward reaching). Further, trunk perturbation magnitude increased throughout the training protocol, with the goal of inducing meaningful trunk movement while allowing the participant to control, at least partially, its displacement. Manual assistance at the pelvis was also gradually implemented to substitute the more stable RobUST constant assistive force. Additionally, training progression included the increased volume of dynamic tasks compensated by a decrease of steady standing time within a session. These points of training progression and the related time course were tailored to the characteristics of research participants and their responses to training (e.g., consistently performing successful attempts of the proposed tasks; subjective feedback from the participant in relation to fatigue and comfort in attempting and performing the proposed tasks), which were reviewed approximately on a weekly basis by the study team. Seated resting periods occurred when requested by the individuals.

### 2.6. Statistical Analysis

Statistical analysis was performed in SAS 9.4 (SAS Inc., Cary, NC, USA) and JASP 0.19 (Amsterdam, The Netherlands). Normal distribution of the data was tested using the Shapiro–Wilk test. The effect of robotic postural stand training (Post vs. Pre training) on the sitting postural control outcomes considered in this study was analyzed within each motor task (steady sitting, self-initiated trunk movements, NRS) and scES condition (i.e., with or without Sit-scES). The NRS ordinal scale scores of the four trunk items considered in this study were assessed by the Wilcoxon signed-rank test. For all other variables, the effect of robotic postural stand training was assessed either by the paired *t*-test or Wilcoxon signed-rank test, depending on the normality distribution outcome. All tests were 2-sided, and the significance level was set as *p* < 0.05. *p*-values < 0.20 are mentioned in the text as trends. The magnitude of robotic postural stand training-promoted changes (Post vs. Pre) on sitting postural control outcomes was also assessed by Cohen’s effect size (ES) [30]. ES values lower than 0.20 were considered negligible, between 0.20 and 0.49 as small, between 0.50 and 0.79 as medium, and equal to or greater than 0.80 as large.

## 3. Results

### 3.1. Steady Sitting Postural Control

**Figure 3:**
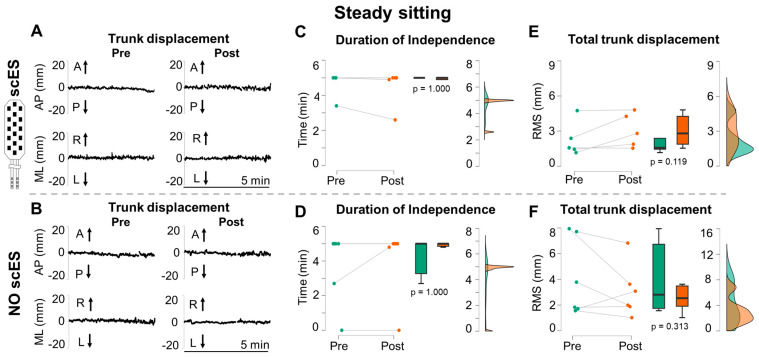
**Steady sitting assessment.** Representative trunk center of mass (CM) antero-posterior (AP) and medio-lateral (ML) displacement collected from participant A96 during 5 min steady sitting attempts performed before (Pre) and after (Post) robotic postural stand training (**A**) with Sit-scES and (**B**) without stimulation (A: anterior; P: posterior; R: right; L: left). Duration of independent sitting and total trunk CM displacement quantified by root mean square (RMS) are reported for the 5 min steady sitting attempts with Sit-scES (**C**,**E**) and without stimulation (**D**,**F**). Pre (green) and Post (red) training data are reported as individual datapoints (*n* = 5 with Sit-scES; *n* = 6 without scES), as box plots (black bold line indicates the sample median; hinges indicate the 25th and 75th quantile; whiskers define the 1.5 interquartile ranges beyond the hinges), and as data density estimates defined by a Gaussian kernel method [31]. The data density estimates are truncated to exclude visualization of negative values. *p* values of the Pre vs. Post comparison for the considered outcomes are also reported.

Research participants demonstrated periods of independent sitting without marked displacement of trunk CM both with and without Sit-scES (Figure 3A and Figure 3B, respectively).

On average, the duration of independent sitting was not different between Post and Pre training, both with Sit-scES (*p* = 1.000; ES: 0.19) and without stimulation (*p* = 1.000; ES: 0.17). Independent sitting was generally maintained for the entire 5 min duration during Pre and Post robotic postural stand training. Exceptions were participant A101 (Figure 3C,D), who achieved some periods of independence with Sit-scES and no independence without spinal stimulation, and B07, who achieved some periods of independence without scES (Figure 3D). Trunk displacement during the steady sitting task (Figure 3E,F) showed a non-significant trend (*p* = 0.119; ES: 0.55) of increasing (i.e., impairment) after training with Sit-scES and no training-induced adaptations without scES (*p* = 0.313; ES: 0.40).

#### 3.1.1. Self-Initiated Trunk Movements

**Figure 4:**
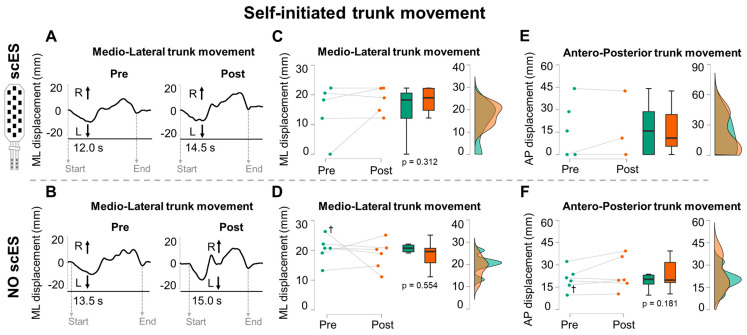
**Self-initiated sitting trunk movements**. Representative trunk center of mass (CM) medio-lateral (ML) displacement (R: right; L: left) collected from participant B45 during self-initiated ML trunk movement attempts performed before (Pre) and after (Post) robotic postural stand training (**A**) with Sit-scES and (**B**) without stimulation. The start and end of the attempts are identified by the respective vertical grey dashed arrows. Peak-to-peak distance covered by the trunk CM is reported for ML attempts (**C**,**D**) and antero-posterior (AP) attempts (**E**,**F**) performed with and without Sit-scES. (**C**–**F**) Pre (green) and Post (red) training data are reported as individual datapoints (*n* = 5 with Sit-scES; *n* = 6 without scES), as box plots (black bold line indicates the sample median; hinges indicate the 25th and 75th quantile; whiskers define the 1.5 interquartile ranges beyond the hinges), and as data density estimates defined by a Gaussian kernel method [31]. The data density estimates are truncated to exclude visualization of negative values. *p* values of the Pre vs. Post comparison for the considered outcomes are also reported. Individual data of Participant A101, who required manual assistance to achieve steady sitting without scES, are identified with a cross (†) (**D**,**F**). During AP attempts with Sit-scES (**E**), data from participants A82 and B45 was not properly collected and could not be used for analysis; thus, statistical analysis for this comparison was not computed.

During self-initiated movement tasks, participants began each trial from a steady, centered sitting position, then attempted to cover with their trunk the largest distance in the cardinal directions considered for the task (e.g., medio-lateral; Figure 4A,B), and finally aimed to regain the centered position without any external assistance. Robotic postural stand training did not influence ML trunk movement performance, as indicated by the Post versus Pre training difference in ML trunk CM displacement with Sit-scES (*p* = 0.312; ES: 0.47; Figure 4C) and without scES (*p* = 0.554; ES: 0.39; Figure 4D). Also, robotic postural stand training promoted a non-significant trend (*p* = 0.181; ES: 0.35) of increasing AP trunk CM displacement without scES (Figure 4F).

#### 3.1.2. Neuromuscular Recovery Scale

**Figure 5:**
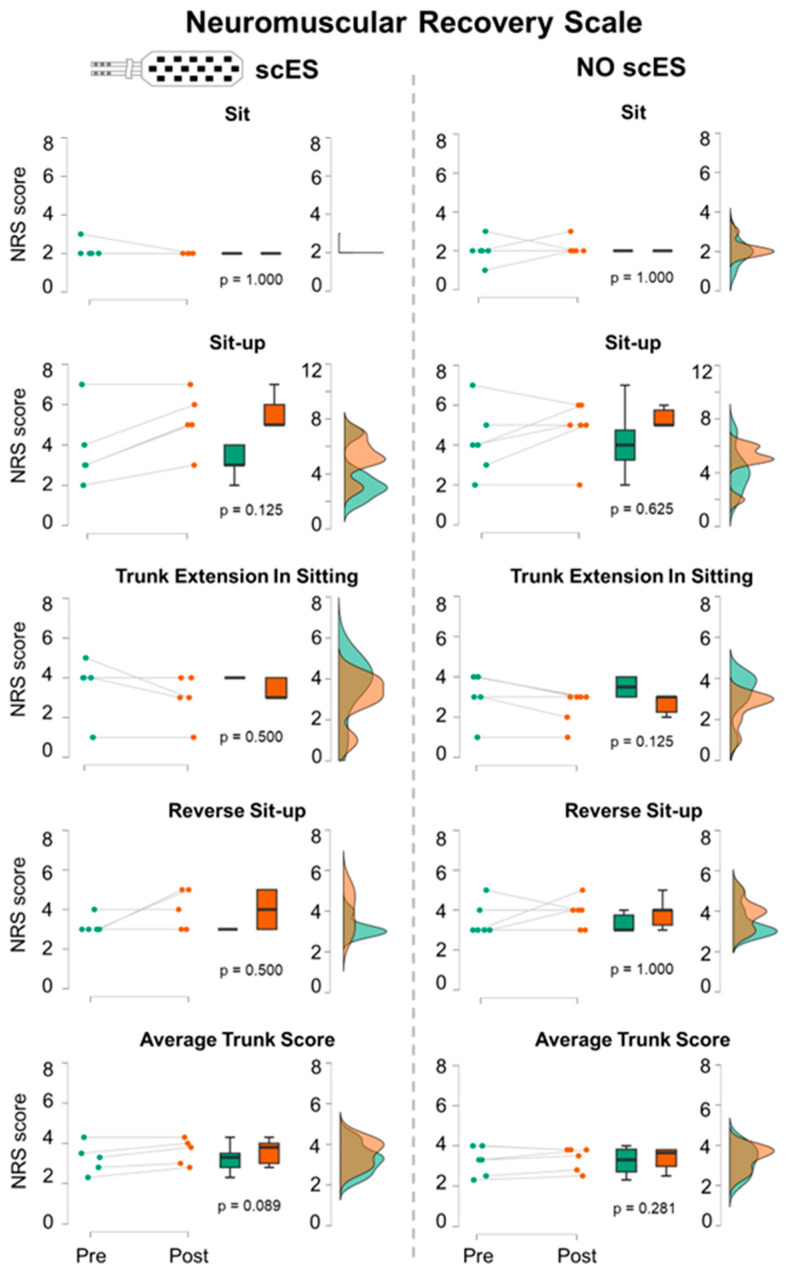
**Neuromuscular Recovery Scale.** Scores of trunk-related items (Sit, Sit-up, Trunk Extension in Sitting, Reverse Sit-up) and the average trunk score (i.e., average score across these four items) are reported for attempts with Sit-scES (**left** panels) and without stimulation (**right** panels). Pre (green) and Post (red) training data are shown as individual datapoints (*n* = 5 with Sit-scES; *n* = 6 without scES), as box plots (black bold line indicates the sample median; hinges indicate the 25th and 75th quantile; whiskers define the 1.5 interquartile ranges beyond the hinges), and as data density estimates defined by a Gaussian kernel method [31]. *p* values of the Pre vs. Post comparison for the considered outcomes are also reported.

Robotic postural stand training did not have a significant impact on the NRS score of trunk-related items (Figure 5). The average trunk score (i.e., average score across the four trunk-related items) showed a non-significant trend (*p* = 0.089; ES: 0.57) of increasing (i.e., improving) after training when trunk function was tested with Sit-scES. This trend was supported by the positive Post vs. Pre trend indicated by the Sit-up task (*p* = 0.125). When performing the NRS without scES, no training effect was observed on the average trunk score (*p* = 0.281; ES: 0.15). Also, a negative trend (*p* = 0.125) was found after training during the trunk extension in sitting.

## 4. Discussion

The robotic postural stand training protocol with scES that led to relevant trunk control improvements in upright standing position did not promote statistically significant changes or large effects in any of the sitting trunk control outcomes considered for analysis. Trends (*p* < 0.2) of improved sitting postural control were observed for self-initiated antero-posterior trunk displacement without scES as well as NRS sit-up and average trunk control with Sit-scES. Conversely, trunk displacement during stable sitting with Sit-scES and NRS trunk extension without scES tended to be impaired after training. The lack of substantial positive transfer effects of robotic postural stand training on sitting trunk control is briefly discussed below, with a particular focus on the neuroplastic potential of the nervous system for motor recovery after SCI and some of its key determinants.

### 4.1. Task Specificity in Training-Induced Neural Plasticity for Motor Recovery

The neuroplastic potential of the mammalian nervous system for motor control is expressed, to a significant degree, as the function of the sensory-motor features repeatedly practiced after SCI [32]. This study presents an interesting paradigm, in which steady and dynamic trunk control tasks assessed in a seated position (Figure 3, Figure 4 and Figure 5) were similar to motor tasks that were repeatedly practiced in an upright standing position. For example, trunk stability was trained over a period of months, with the participants attempting to maintain upright steady standing with free hands and constant robotic assistance for pelvic control. Training progression included the progressive removal of external assistance at the trunk (i.e., robotic assistance as needed) and the inclusion of arm reaching movements for self-posture perturbation as well as controlled perturbations applied at the trunk toward the four cardinal directions [21]. Also, dynamic trunk control was practiced in standing with free hands by asking the participants to perform, among others, large antero-posterior and medio-lateral trunk movements.

To date, the prevailing view is that activity-based training promotes functional recovery and neuroplasticity through intense, task-specific training that provides proper sensory inputs to the spinal circuitries (i.e., appropriate sensory-motor therapy) [33,34,35]. For example, in one of our proof-of-principle studies, we showed that standing ability improved after a block of stand training with scES in all four individuals with motor complete SCI; however, the subsequent block of step training with scES impaired standing in three of the four participants [13]. This is consistent with previous SCI animal model studies reporting that stand training can impair the ability to step [36] and to perform an untrained motor task that involves lower limb flexion [37]. Conversely, the concurrent practice of standing and stepping with scES appears beneficial for the recovery of both motor tasks [14,17]. This is in line with animal model studies suggesting that a certain degree of variability within the trained motor task (i.e., variability of the step cycle kinematics; training stepping in different directions) may be beneficial for motor learning [38,39]. Taken together, the findings reported in the literature may support the view that the trunk motor tasks practiced during robotic postural stand training are not as stereotypical (i.e., several blocks of a given motor task) and/or as different from those tested in sitting, to explain the lack of substantial positive effect observed in this study. Also, in a different neurological population (individuals with stroke) and when a robotic device guides the movement pattern (i.e., robotic-assisted gait training), individual-specific joint trajectories can improve cardiometabolic responses during gait training compared to generalized joint trajectories [40]. This may suggest that individual-specific characteristics can also interact with task-specific sensory-motor information and therefore may be an additional variable to take into consideration for future controlling algorithms of rehabilitative robotic devices [41].

### 4.2. Posture Specificity as a Conceivable Determinant of Training-Induced Neural Plasticity

The role of peripheral sensory information in the context of spinal motor control and learning has long been investigated and reported [42,43,44]. Inputs deriving from a variety of peripheral sensory receptors can interact with the spinal circuitries responsible for motor control to initiate and modulate motor pattern generation. Further evidence supports the view that these motor control mechanisms are not solely related to locomotion. For example, enhanced sitting trunk control was promoted by targeted spinal cord transcutaneous stimulation, while balance was self-perturbated by rapid upper limb movements [4]. This was interpreted as the spinal postural networks re-enabled by spinal stimulation being able to perform enhanced sensory processing and related motor control. As mentioned above, repetitive sensory inputs to the spinal networks also appear to be a key determinant of neural plasticity and related motor recovery after SCI (i.e., appropriate sensory motor therapy) [33,34,35].

In the present study, sensory inputs repeatedly processed by the spinal circuitry during robotic postural stand training are at least partially different than those received during the sitting trunk control assessments because of the body positions maintained during the two tasks. For example, the hip joint was extended during robotic postural stand training, while it was flexed during the sitting assessments. Thus, hip joint receptors and mechanoreceptors related to muscles crossing the hip joint (i.e., muscle spindles of iliopsoas), among others, were conceivably providing different inputs to the spinal cord. A second relevant aspect is related to the different pelvic tilt maintained between robotic postural stand training and sitting assessments. During robotic postural stand training, RobUST provided constant assistance aimed at maintaining appropriate neutral pelvic tilt (as well as centered pelvis position) [20,21]. Conversely, pelvic tilt was not assisted in sitting, resulting in a more kyphotic (C-shaped) trunk posture and posterior pelvic tilt [4,18,45]. Studies in non-injured adults have reported increased activation in trunk extensor muscles during anterior/neutral pelvic tilt from posterior pelvic tilt [46]. Therefore, a neutral pelvic position may facilitate activation of trunk muscles even if achieved via external assistance (i.e., RobUST).

The importance of body posture for sensory inputs on motor control is also exemplified by a case study of an individual with chronic C3 sensory motor complete SCI. This individual was able to generate a variety of motor patterns with different body segments, such as push-ups from a prone position, trunk extension from a seated position, and hip-bridge from a supine position, only when specific joints alignments were implemented and related sensory information was received by the spinal cord [47]. This ability was gained after long-term activity-based recovery training [48]. Conversely, in non-optimal or sensory-deprived positions (i.e., supine position with lower limbs extended), no ability to use upper extremities and no voluntary movement of lower extremities were observed.

### 4.3. Limitations

The limited access to participants that undertook this study during the COVID-19 pandemic resulted in some missing data (e.g., Sit-scES data for participant B07). This pilot study was not designed to provide generalized results for the SCI population, given the small sample size with heterogeneous characteristics, including the amount of previous activity-based training with scES for sitting (Table 1) and standing [21], and the lack of a control group. Also, pelvic tilt was characterized by real-time and off-line (i.e., video recordings) visual inspection rather than being quantified by kinematics. This was partly due to the need to optimize the placement and number of reflective markers for the control of RobUST rather than for kinematic analysis. Future investigations on the present topic should also include and examine more detailed biomechanical and physiological variables. For example, the kinematic analysis of specific trunk segments by curvature and inclination outcomes may highlight adaptations that would not be reflected by more global variables such as trunk CM [4,18]. Assessment of trunk muscle activation (if properly discriminated from spinal cord electrical stimulation artifacts) as well as cardiovascular outcomes could also contribute to explain the difference in postural control adaptations before and after intervention and between different body positions, such as sitting and standing.

### 4.4. Conclusions

In summary, the robotic postural stand training protocol with Stand-scES that led to relevant trunk control improvements in upright standing position did not promote statistically significant changes or large effects in any of the sitting trunk control outcomes considered for analysis in a population of high-level motor complete SCI individuals. Future studies should investigate, prospectively, whether, and to what extent, posture specificity plays a role in determining activity-based training-induced neural plasticity and motor recovery. Future studies may also investigate how the concurrent practice of robotic postural training with scES in standing and sitting would affect trunk control in sitting, as this approach may provide a positive balance between specificity and variability of the sensory-motor inputs for motor recovery.

## Figures and Tables

**Table 1:** Characteristics of the research participants. Pub ID: publication identifier; F: female; M: male; level of injury: neurological level of the lesion by AIS (American Spinal Injury Association (ASIA) Impairment Scale); C: cervical; T: thoracic; scES: spinal cord epidural stimulation; sitting trunk practice time: the total duration individuals practiced sitting postural control with scES prior to the beginning of this study.

Pub ID	Age (Yrs)	Sex	Time since Injury (Yrs)	Level of Injury	AIS	Time since scES Implant (Yrs)	Sitting Trunk Practice Time (Hrs)
A96	29	F	5.3	C4	A	1.7	268
A101	33	M	4.2	C3	A	1.6	260
A82	37	M	8.8	C4	A	1.2	601
B45	36	M	9.3	C7	B	0.3	3
B07	35	M	14.4	T2	B	11.0	-
B23	38	M	9.4	C4	B	6.1	25

## Data Availability

Data that support the findings herein reported will be made available through material transfer agreement upon reasonable request.

## Supplementary Materials

The following supporting information can be downloaded at: https://www.mdpi.com/article/10.3390/jcm13154309/s1, Figure S1. Epidural stimulation parameters to facilitate sitting postural control; Figure S2. Stimulation parameters to facilitate standing during robotic postural stand training.
