# Supplementary material for: Effects of Robotic Postural Stand Training with Epidural Stimulation on Sitting Postural Control in Individuals with Spinal Cord Injury: A Pilot Study
Source: J Clin Med. 2024 Jul 24;13(15):4309. doi: 10.3390/jcm13154309 (PMC11313204; doi:10.3390/jcm13154309)
Supplement: Supplementary file 1 [file jcm-13-04309-s001.zip › jcm-3107089-supplementary.pdf]

Supplementary Information

Pub ID	Sit-scES parameters
A96	**P1.** 5- 11- // 0+ 2+ 3+ 4+ 6+ 8+ 9+ 10+ 13+ 14+ 15+<br>11.6 mA; 680 µs; 65 Hz
A101	**P1.** 0- 5- 11- // 2+ 3+ 4+ 7+ 8+ 9+ 10+ 13+ 15+<br>7.7 mA; 700 µs; 45 Hz<br><br>**P2.** 5- 11- // 2+ 3+ 4+ 7+ 8+ 9+ 10+ 13+ 14+ 15+<br>5.0 mA; 700 µs; 45 Hz
A82	**P1.** 0- 5- 11- // 1+ 2+ 3+ 4+ 6+ 7+ 8+ 9+ 10+ 12+ 13+ 14+ 15+<br>5.6 mA; 800 µs; 40 Hz<br><br>**P2.** 0- 5- 11- // 1+ 2+ 6+ 7+ 12+ 13+ ; 3.2 mA; 800 µs; 40 Hz<br><br>**P3.** 0- 1- // 3+ 4+ 9+ 10+ 14+ 15+ ; 3.2 mA; 800 µs; 40 Hz
B45	**P1.** 0- 5- 11- // 1+ 2+ 7+ 12+ 13+ ; 9.1 mA; 700 µs; 23 Hz<br><br>**P2.** 4- 10- 15- // 1+ 2+ 6+ 7+ 8+ 12+ 13+ ; 7.6 mA; 300 µs; 115 Hz<br><br>**P3.** 0- 5- 11- // 1+ 6+ 12+ ; 8.5 mA; 700 µs; 19 Hz
B07	No Sit-scES
B23	**P1.** 5- // 2+ 6+ 7+ 13+ ; 2.5 V; 450 µs; 30 Hz<br><br>**P2.** 0- 5- 11- // 6+ 7+ ; 2.5 V; 450 µs; 30 Hz<br><br>**P3.** 5- 6- // 2+ 3+ 7+ 8+ 13+ 14+ ; 2.8 V; 450 µs; 30 Hz

Electrode array

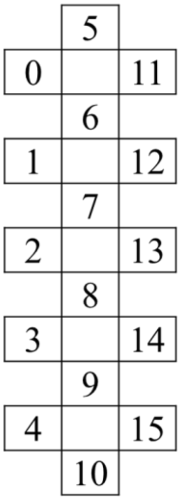

Figure S1. Epidural stimulation parameters to facilitate sitting postural control. Multiple stimulation programs (P1 to P3) were applied to the research participants with independent frequencies (A101, A82, B45) or in an interleaved fashion (B23), depending on the stimulator unit characteristics. Active contacts of the 16-electrode array are listed as cathodes (-) or anodes (+). Stimulation pulse width, frequency, and representative amplitude are also reported for each stimulation program; stimulation amplitude was adjusted throughout each session if needed.

Pub ID	Stand-scES parameters
A96	**P1.** 0- 5- 11- // 1+ 6+ 7+ 12+ ; 3.2 mA; 500 µs; 19 Hz
	**P2.** 4- 9- 15- // 1+ 2+ 3+ 7+ 12+ 13+ 14+ ; 6.8 mA; 700 µs; 19 Hz
	**P3.** 1- 7- 12- // 0+ 6+ 11+ ; 11.2 mA; 700 µs; 23 Hz
A101	**P1.** 2- 3- 4- 7- 13- // 0+ 5+ 11+ ; 2.6 mA; 700 µs; 25 Hz
	**P2.** 3- 4- 10- // 0+ 1+ 2+ 7+ 8+ ; 3.5 mA; 750 µs; 17 Hz
	**P3.** 0- 1- 6- 11- // 2+ 7+ 13+ ; 2.6 mA; 800 µs; 25 Hz
	**P4.** 0- 1- 6- 11- // 2+ 7+ 13+ ; 2.6 mA; 900 µs; 25 Hz
A82	**P1.** 2- 3- 13- 14- // 0+ 5+ 6+ 11+ ; 3.1 mA; 700 µs; 30 Hz
	**P2.** 7- 8- // 0+ 4+ 5+ 6+ 9+ 10+ 11+ 15+ ; 2.0 mA; 700 µs; 30 Hz
B45	**P1** *(Early)*. 2- 8- 13- // 0+ 1+ 6+ 7+ 12+ ; 3.8 mA; 890 µs; 35 Hz
	**P2** *(Early)*. 8- // 9+ 10+ 14+ 15+ ; 2.0 mA; 500 µs; 35 Hz
	**P1** *(Mid & Late)*. 2- 7- 13- // 0+ 1+ 6+ 12+ ; 3.5 mA; 890 µs; 35 Hz
	**P2** *(Mid & Late)*. 10- 15- // 1+ 2+ 3+ 4+ 7+ 8+ 9+ 12+ 13+ 14+<br>4.4 mA; 500 µs; 35 Hz *(Mid)* - 52 Hz *(Late)*
B07	**P1.** 0- 1- 8- 11- 12- // 4+ 5+ 6+ 9+ 10+ 15+ ; 4.5 mA; 1000 µs; 22 Hz
	**P2.** 3- 8- 14- // 1+ 4+ 7+ 9+ 12+ 15+ ; 2.0 mA; 1000 µs; 22 Hz
	**P3.** 2- 8- 13- // 0+ 4+ 5+ 6+ 9+ 11+ 15+ ; 2.1 mA; 1000 µs; 22 Hz
	**P4.** 2- 3- 8- 13- 14- // 0+ 1+ 4+ 5+ 6+ 7+ 9+ 10+ 11+ 12+ 15+<br>4.5 mA; 1000 µs; 22 Hz
B23	**P1.** 5- 6- 11- // 1+ 2+ 7+ 8+ 12+ 13+ ; 2.1 V; 450 µs; 40 Hz
	**P2.** 0- // 2+ 3+ 5+ 6+ 7+ 11+ 12+ ; 2.5 V; 450 µs; 40 Hz
	**P3.** 1- 2- 6- 7- // 0+ 5+ ; 1.0 V; 450 µs; 40 Hz

Electrode array

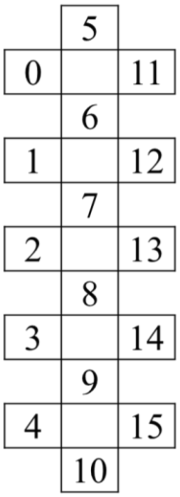

Figure S2. Stimulation parameters to facilitate standing during robotic postural stand training. Multiple stimulation programs (P1 to P4) were applied to the research participants in an interleaved fashion (B23), or with independent frequencies (A96, A101, A82, B45, B07), depending on the stimulator unit characteristics. Active contacts of the 16-electrode array are listed as cathodes (-) or anodes (+). Stimulation pulse width, frequency, and representative amplitude are also reported for each stimulation program; stimulation amplitude was adjusted throughout each session if needed. Stimulation parameters of participant B45 were modified throughout robotic postural stand training, and are reported here for representative portions of training (early, mid and late part of training).
